# The prevalence of the term subluxation in chiropractic degree program curricula throughout the world

**DOI:** 10.1186/s12998-018-0191-1

**Published:** 2018-07-02

**Authors:** Matthew F. Funk, Aric J. Frisina-Deyo, Timothy A. Mirtz, Stephen M. Perle

**Affiliations:** 1 University of Bridgeport College of Chiropractic, Bridgeport, CT USA; 2 Department of Secondary and Physical Education, Bethune-Cookman University, Daytona Beach, FL USA; 3 Discipline of Chiropractic, School of Health Professions, Murdoch University, Murdoch, WA Australia

**Keywords:** Chiropractic, Professional education, Curriculum, Philosophy, Accreditation, Licensure, Evidence-based practice

## Abstract

**Background:**

The subluxation construct generates debate within and outside the profession. The International Chiropractic Education Collaboration, comprised of 10 chiropractic programs outside of North America, stated they will only teach subluxation in a historical context. This research sought to determine how many chiropractic institutions worldwide still use the term in their curricula and to expand upon the previous work of Mirtz & and Perle.

**Methods:**

Forty-six chiropractic programs, 18 United States (US) and 28 non-US, were identified from the World Federation of Chiropractic Educational Institutions list. Websites were searched by multiple researchers for curricular information September 2016–September 2017. Some data were not available on line, so email requests were made for additional information. Two institutions provided additional information. The total number of mentions of subluxation in course titles, technique course (Tech) descriptions, principles and practice (PP) descriptions, and other course descriptions were reported separately for US and non-US institutions. Means for each category were calculated. The number of course titles and descriptions using subluxation was divided by the total number of courses for each institution and reported as percentages.

**Results:**

Means for use of subluxation by US institutions were: Total course titles = .44; Tech = 3.83; PP = 1.50; other = 1.16. For non-US institutions, means were: Total course titles = .07; Tech = .27; PP = .44; other = 0. The mean total number of mentions was 6.94 in US vs. 0.83 in non-US institutions. Similarly, the mean course descriptions was 6.50 in US vs. 0.72 in non-US institutions.

**Conclusions:**

The term subluxation was found in all but two US course catalogues. The use of subluxation in US courses rose from a mean of 5.53 in 2011 to 6.50 in 2017. US institutions use the term significantly more frequently than non-US. Possible reasons for this were discussed. Unscientific terms and concepts should have no place in modern education, except perhaps in historical context. Unless these outdated concepts are rejected, the chiropractic profession and individual chiropractors will likely continue to face difficulties integrating with established health care systems and attaining cultural authority as experts in conservative neuro-musculoskeletal health care.

## Background

Professionalization and development of cultural authority can involve changing educational curricula as has occurred in the medical profession [1]. These changes in curricula can be the result of scientists defining what is and is not science. This type of work is what is known in sociology as boundary-work. Boundary-work leads to a division within a profession where scientists exclude pseudoscience and thus excludes some as outsiders [2].

Boundary-work appears to be occurring in the chiropractic profession [2, 3]. The boundary-work may be separating the scientific academics from the practicing field and more vitalistic academics [2]. The developing boundary appears to be the often cited crossroads for the profession which centers on the issue of the subluxation [4–37]. While the vitalistic academics may believe that there is adequate scientific evidence for the subluxation that does not appear to be true [24, 28].

This controversy has resulted in The International Chiropractic Education Collaboration ICEC issuing a position statement that the chiropractic programs would only teach about subluxation in a historical context. This collaboration included ten chiropractic programs outside of North America [38]. 

Previously Mirtz and Perle [19] found only three English-language chiropractic programs in North America avoided mentioning the subluxation in their course titles or descriptions. Given the inability to actually determine what specifically is taught throughout the chiropractic curricula without a statement such as that from ICEC, counting the prevalence of the term subluxation in the course title or description seems a reasonable method to ascertain the position of other chiropractic programs with regards to the subluxation construct. The development of this international position statement on the teaching of subluxation within chiropractic training programs we believe showed a need to expand upon the work of Mirtz and Perle to an assessment of the curricula of chiropractic programs around the world. Thus, the purpose of this study was to expand upon the previous work of Mirtz and Perle to determine the prevalence of the term subluxation in the course tittles or descriptions in all chiropractic training programs around the world for which we could find information.

## Methods

### Sample population and eligibility criteria

Chiropractic colleges were identified from the World Federation of Chiropractic Educational Institutions list [39]. Websites were accessed by multiple researchers with the most recent catalogues and course listings retrieved from available institutions. If researchers were unable to locate the required information, an email was sent either directly through website to the institution or to an individual associated with the institution. Available data were reported. 

Only course listings from 2015 to 2016 or 2016–2017 were used. Institutions with multiple campuses were considered separate entities if a campus-specific catalogue was available. A list of 46 chiropractic degree granting institutions was established including diploma, Bachelor of Science (BS), Master of Science (MS) and Doctor of Chiropractic (DC) programs. Of the 46 programs, 36 full course descriptions and 10 course title-only listings were retrieved either through Adobe Acrobat portable document format or through the college’s web-based platform. Table 1 shows the institutions from which either catalogues or course listings were retrieved.

**Table 1:** Chiropractic degree-granting institutions

Central Queensland University	Australia
Macquarie University	Australia
Murdoch University	Australia
RMIT University	Australia
Centro Universitario Feevale	Brazil
Universidade Anhembi Morumbi	Brazil
Canadian Memorial Chiropractic College	Canada
Universite du Quebec a Trois-Rivieres	Canada
Universidad Central de Chile	Chile
Syddansk Universitet Odense (University of Southern Denmark)	Denmark
IFEC Campus de Paris et de Toulouse	France
Tokyo College of Chiropractic	Japan
International Medical University	Malaysia
Universidad Estatal del Valle de Ecatepec	Mexico
Universidad Estatal del Valle de Toluca	Mexico
Universidad Veracruzana	Mexico
New Zealand College of Chiropractic	New Zealand
Hanseo University	Republic of Korea
Durban University of Technology	South Africa
University of Johannesburg	South Africa
Barcelona College of Chiropractic	Spain
Madrid College of Chiropractic - RCU	Spain
Skandinaviska Kiropraktorhogskolan (Scandinavian College of Chiropractic)	Sweden
University of Zurich	Switzerland
Bahcesehir University - Chiropractic Program	Turkey
AECC University College	United Kingdom
McTimoney College of Chiropractic	United Kingdom
University of South Wales - Welsh Institute of Chiropractic	United Kingdom
Cleveland Chiropractic College	United States of America
D’Youville College	United States of America
Keiser University	United States of America
Life Chiropractic College West	United States of America
Life University	United States of America
Logan University	United States of America
National University of Health Sciences	United States of America
New York Chiropractic College	United States of America
Northwestern Health Sciences University	United States of America
Palmer College of Chiropractic (Davenport Campus)	United States of America
Palmer College of Chiropractic (Florida Campus)	United States of America
Palmer College of Chiropractic (West Campus)	United States of America
Parker University	United States of America
Sherman College of Chiropractic	United States of America
Southern California University of Health Sciences	United States of America
Texas Chiropractic College	United States of America
University of Bridgeport	United States of America
University of Western States	United States of America

### Data extraction

The search function of Adobe Acrobat reader program or Google Chrome web browser was used to search course descriptions and titles for “subl” denoting the use of the words subluxate, subluxated, subluxation (English and French), sublussazione (Italian), subluxación (Spanish), subluxação (Portuguese), subluksation (Danish), and subluksasyon (Turkish). Mirtz and Perle [19] used a similar process for North American English-language programs. Use of this term was sorted into categories as seen in Table 2. Courses which combined elements of chiropractic principles and practice or methods with a technique lab portion were considered technique courses to differentiate between applied chiropractic techniques and philosophy courses. If the term “subl” appeared multiple times in a single title or course description it was counted only once to prevent double counting. If an institution had more than one chiropractic degree granting program, the higher-level degree (MS or BS) was used for data collection. Data were collected from September 2016–September 2017 and entered into Google Sheets with two researchers independently reviewing each data point. Any discrepancy between researchers was resolved by discussion and thorough review. If researchers did not agree following recount, a third researcher was brought in to resolve the issue.

**Table 2:** Data Extraction Categories

Total number of times mentioned	
Subluxation mentioned in course description	
Subluxation mentioned in course title	
Subluxation mentioned in technique course description	
Subluxation mentioned in chiropractic principles and practice course description	
Subluxation mentioned in other courses	
Total number of courses	

### Data analysis

Totals for each institution were calculated as the sum of mentions of the term subluxation in course titles, technique course descriptions, principles and practice descriptions, and other course descriptions. Mirtz and Perle’s earlier study provides data from United States (US) institutions which allows for timewise comparisons for those institutions [19]. Thus we calculated means for total number of times mentioned, times mentioned in course titles, course descriptions, technique courses, principles and practices courses and other courses for institutions in the US and outside of the US. Following data collection, the number of course titles and descriptions using the term “subl” was divided by the total number of courses for each institution and reported as percentages.

## Results

### US institutions

All 18 US institutions had websites containing curricular information. Since the three Palmer campuses share one catalogue, 16 different web-based catalogues were studied. Complete course titles and descriptions were available for all US institutions. The US institutions with the greatest number of mentions of the term subluxation in courses and course titles were Life University with 25, Sherman College of Chiropractic with 17 and Palmer College of Chiropractic-Florida with 16. When all three Palmer institutions were combined, subluxation was mentioned 29 times. National University of Health Sciences and Southern California University of Health Sciences did not mention the term at all. Table 3 depicts the total number of times subluxation was used, total in course descriptions and total in course titles for US institutions. The percentage of times subluxation was used in course descriptions is also provided in Table 3. Data from the 2011 study are provided in parentheses for comparison to current data. The four most prevalent mentions of subluxation were found in the catalogues of Parker University (19.14%), Palmer College of Chiropractic-Florida (16.67%), Sherman College of Chiropractic (12.82%) and Life University (12.69%). The aggregated mean mentions of subluxation for all US institutions was 6.94. The mean mentions in course descriptions was 6.50.

**Table 3:** Use of term subluxation in catalogues of United States chiropractic degree programs

Doctor of Chiropractic Degree Program	Number of times subluxation mentioned	Number in course descriptions	Total number of courses	Percent in course descriptions
Cleveland Chiropractic College	5 (6)^a^	5 (4)	81 (79)	6.17 (5.06)
D’Youville College	7 (6)	7 (2)	88 (46)	7.95 (4.34)
Keiser University	1 (N/A)	1 (N/A)	73 (N/A)	1.36 (N/A)
Life Chiropractic College West	10 (14)	9 (8)	113 (92)	7.96 (8.70)
Life University	25 (28)	24 (24)	189 (146)	12.69 (16.44)
Logan University	5 (12)	5 (5)	95 (96)	5.26 (5.21)
National University of Health Sciences	0 (0)	0 (0)	85 (75)	0 (0)
New York Chiropractic College	4 (5)	4 (3)	109 (72)	3.67 (4.16)
Northwestern Health Sciences University	5 (7)	5 (2)	105 (85)	4.76 (2.35)
Palmer College of Chiropractic (Davenport Campus)	9 (55)^b^	8 (6)	83 (72)	9.64 (8.33)
Palmer College of Chiropractic (Florida Campus)	16 (55)^b^	14 (10)	84 (44)	16.67 (22.72)
Palmer College of Chiropractic (West Campus)	4 (55)^b^	4 (5)	91 (78)	4.39 (6.41)
Parker University	10 (16)	9 (6)	47 (74)	19.14 (8.11)
Sherman College of Chiropractic	17 (53)	15 (11)	117 (86)	12.82 (12.80)
Southern California University of Health Sciences	0 (0)	0 (0)	72 (67)	0 (0)
Texas Chiropractic College	1 (2)	1 (2)	66 (66)	1.51 (3.03)
University of Bridgeport	1 (6)	1 (1)	75 (74)	1.33 (1.35)
University of Western States	5 (6)	5 (5)	117 (104)	4.27 (4.80)
Means	6.94	6.50 (5.53)	93.89 (79.76)	6.64% (6.69%)

^a^ Data in parentheses are from Mirtz and Perle (2011)

^b^ Data for all three Palmer campuses were combined in Mirtz and Perle (2011)

The use of subluxation in each course category is shown in Table 4. The term subluxation in course titles was uncommon, with a mean of 0.44. The number of times the term was used in technique course descriptions, principles and practices descriptions and in other course descriptions is presented along with data from 2011 in parentheses for comparison. Life University used subluxation in 11 technique courses, followed by Palmer College-Florida and Sherman College with 9 each. The mean mentions for all US institutions in technique classes was 3.83, 1.50 for principles and practices, and 1.16 in other classes. Subluxation showed up 11 times in other classes at Life University, including 4 times in diagnosis classes, 4 times in analysis classes and once each in public health, biochemistry and practice management course descriptions.

**Table 4:** Specific usage of the term subluxation in United States chiropractic degree program

Chiropractic Program	Number in course titles	Number in technique course descriptions	Number in principles and practice descriptions	Number in other course descriptions
Cleveland Chiropractic College	0 (0)^a^	3 (2)	2 (2)	0
D’Youville College	0 (0)	6 (2)	1 (0)	0
Keiser University	0 (N/A)	0 (N/A)	1 (N/A)	0 (N/A)
Life Chiropractic College West	1 (1)	6 (5)	3 (1)	0
Life University	1 (1)	11 (14)	2 (1)	11
Logan University	0 (0)	2 (2)	3 (3)	0
National University of Health Sciences	0 (0)	0 (0)	0 (0)	0
New York Chiropractic College	0 (0)	2 (1)	2 (2)	0
Northwestern Health Sciences University	0 (0)	4 (1)	1 (0)	0
Palmer College of Chiropractic (Davenport)	1 (0)	4 (4)	2 (2)	2
Palmer College of Chiropractic (Florida)	2 (4)	9 (2)	2 (0)	3
Palmer College of Chiropractic (West)	0 (0)	2 (2)	2 (3)	0
Parker University	1 (0)	7 (4)	1 (1)	1
Sherman College of Chiropractic	2 (1)	9 (6)	2 (1)	4
Southern California University of Health Sciences	0 (0)	0 (0)	0 (0)	0
Texas Chiropractic College	0 (0)	0 (0)	1 (2)	0
University of Bridgeport	0 (1)	0 (0)	1 (1)	0
University of Western States	0 (0)	4 (3)	1 (2)	0
Means	0.44 (0.47)	3.83 (2.82)	1.50 (1.23)	1.16

^a^ Data in parentheses are from Mirtz and Perle (2011)

### Non-US institutions

There are currently 28 non-US educational institutions offering chiropractic degrees. Most institutions had websites depicting at least course titles by year. Curricular data were obtained from these websites, except for Institut Franco-Européen de Chiropraxie (France) and Madrid College of Chiropractic, Real Centro Universitario (Spain), which provided information after an email request. Several institutions did not have full curricula available. Attempts were made to obtain missing information via direct email or links for more information. Data were not available or incomplete for 10 ten institutions: Universidad Central de Chile (Chile); Tokyo College of Chiropractic (Japan); International Medical University (Malaysia); Universidad Estatal del Valle de Toluca (Mexico); Universidad Estatal del Valle de Ecatepec (Mexico); New Zealand College of Chiropractic (New Zealand); University of Johannesburg (South Africa); Skandinaviska Kiropraktorhogskolan (Sweden); University of Zurich (Switzerland); McTimoney College of Chiropractic (United Kingdom).

Table 5 presents the use of the term subluxation in non-US chiropractic programs. Subluxation was mentioned rarely, with a range of 0–4 times and a mean of 0.83 in course titles plus descriptions. There were few mentions of subluxation in course descriptions (range 0–4 with mean 0.72). The total number of courses is also provided along with a breakdown for BS and MS programs when offered. The total number of courses used to calculate the percentage of subluxation mentions in course descriptions was based on the total for the more advanced degree (MS). Subluxation was mentioned between 0 and 12.50% of course descriptions with a mean of 1.38%.

**Table 5:** Use of term subluxation in catalogues of non-US chiropractic degree programs

Chiropractic Program, Country	Number of times subluxation mentioned	Number in course descriptions	Total number of courses and degree(s) granted	Percent in course descriptions
Central Queensland University, Australia	0	0	24 B.S.; 42 M.S.	0
Macquarie University, Australia	0	0	24 B.S.; 46 M.S.	0
Murdoch University, Australia	0	0	35 B.S.; 37 Honors	0
RMIT University, Australia	0	0	37 B.S. (5 yr.)	0
Centro Universitario Feevale, Brazil	0	0	73 B.S.	0
Universidade Anhembi Morumbi, Brazil	1	0	40 B.S.	0
Canadian Memorial Chiropractic College, Canada	0	0	58 D.C.	0
Universite du Quebec a Trois-Rivieres, Canada	2	2	75 D.C.	2.67
Universidad Central de Chile, Chile^a^			67 B.S.	
Syddansk Universitet Odense, Denmark	4	4	16 B.S.; 32 M.S.	12.50
Institut Franco-Européen de Chiropraxie, France^b^	1	1	166 M.S.	.60
Tokyo College of Chiropractic, Japan^a^			103 D.C.	
International Medical University, Malaysia^a^			59 B.S.	
Universidad Estatal del Valle de Ecatepec, Mexico^a^			61 diploma	
Universidad Estatal del Valle de Toluca, Mexico^a^			68 diploma	
Universidad Veracruzana, Mexico	1	1	62 diploma	1.61
New Zealand College of Chiropractic, New Zealand^a^			33 B.S. (5 yr.)	
Hanseo University, Republic of Korea	4	3	58 D.C.	5.17
Durban University of Technology, South Africa	0	0	30 diploma	0
University of Johannesburg, South Africa^a^			33 diploma	
Barcelona College of Chiropractic, Spain	1	1	32^c^ (5 yr.)	3.12
Madrid College of Chiropractic – RCU, Spain^b^	1	1	52^c^ (5 yr.)	1.92
Skandinaviska Kiropraktorhogskolan, Sweden^a^			60 (5 yr.)	
University of Zurich, Switzerland^a^			41^d^ (6 yr.)	
Bahcesehir University - Chiropractic Program, Turkey	0	0	17 M.S.	0
AECC University College, United Kingdom	0	0	28^e^	0
McTimoney College of Chiropractic, United Kingdom^a^			24 (4 yr); 27^e^ (5 yr)	
University of South Wales - Welsh Institute of Chiropractic, United Kingdom	0	0	36^e^	0
Means	0.83	0.72	52.61	1.38%

^a^ Email sent either directly through website to the institution or to an individual associated with the institution. Available data were reported. Blank spaces indicate corresponding information was not available or provided

^b^ Not from website (data obtained through personal e-mail)

^c^ Senior degree in chiropractic

^d^ Master of Chiropractic Medicine

^e^ Master of Chiropractic

The specific use of the term subluxation in course titles, technique courses, principles and practices courses and other classes are shown in Table 6. Once again, the term was used rarely in non-US institutions, with a mean of 0.08 times in titles, 0.27 times in technique, 0.44 times in principles and practices, and zero times in other classes.

**Table 6:** Specific usage of the term subluxation in non-US chiropractic degree programs

Chiropractic Program	# in course titles	# in technique course descriptions	# in principles and practice descriptions	# in other course descriptions
Central Queensland University	0	0	0	0
Macquarie University	0	0	0	0
Murdoch University	0	0	0	0
RMIT University	0	0	0	0
Centro Universitario Feevale	0	0	0	0
Universidade Anhembi Morumbi	1	0	0	0
Canadian Memorial Chiropractic College	0	0	0	0
Universite du Quebec a Trois-Rivieres	0	0	2	0
Universidad Central de Chile^a^	0			
Syddansk Universitet Odense (University of Southern Denmark)	0	4	0	0
Institut Franco-Européen de Chiropraxie^b^	0	0	1	0
Tokyo College of Chiropractic^a^	0			
International Medical University^a^	0			
Universidad Estatal del Valle de Ecatepec^a^	0			
Universidad Estatal del Valle de Toluca^a^	0			
Universidad Veracruzana	0	1	0	0
New Zealand College of Chiropractic^a^	0			0
Hanseo University	1	0	3	0
Durban University of Technology	0	0	0	0
University of Johannesburg^a^	0			
Barcelona College of Chiropractic	0	0	1	0
Madrid College of Chiropractic – RCU^b^	0	0	1	0
Skandinaviska Kiropraktorhogskolan (Scandinavian College of Chiropractic)^a^	0			
University of Zurich^a^	0			
Bahcesehir University Chiropractic Program	0	0	0	0
AECC University College	0	0	0	0
McTimoney College of Chiropractic^a^	0			
University of South Wales Welsh Institute of Chiropractic	0	0	0	0
Means	0.07	0.27	0.44	0

^a^ Email sent either directly through website to the institution or to an individual associated with the institution. Available data were reported. Blank spaces indicate corresponding information was not available or provided

^b^ Not from website (from personal e-mail)

## Discussion

The subluxation construct has been a contentious topic throughout the history of the chiropractic profession [28]. Adherents of the subluxation construct have argued several rationales for its continued use [22]. These arguments have come from five primary rationales: professional identity, philosophical, technical, legal and accreditation [28]. Each of these rationales will be discussed in context to the subluxation construct and its usage within international programs.

### Subluxation construct – Professional identity rationale

The professional identity rationale argues that the subluxation is what separates the profession from other health care professions. Without the subluxation (and its accompanying philosophy), it is argued that chiropractors would be nothing more than physical therapists who use spinal manipulation [9]. In the US alone, there are 18 chiropractic college programs. This study found that of the chiropractic programs in the US, 88% (*n* = 16) presented subluxation in their college catalogs. Mirtz and Perle [19], in their study of North America English-Language programs, had a similar finding. Yet, of the 19 non-US programs from which data could be gleaned, 47.4% (*n* = 9) mentioned the term in their catalogs. This suggests to us that professional identity driven primarily by the concept of subluxation may be less important in countries outside the US. While we cannot comment on how and why subluxation was included in course titles or descriptions, fewer mentions of subluxation in public documents outside the US suggests that professional identity in non-US countries is not as dependent upon adherence to the subluxation construct as in the US.

### Subluxation construct – Philosophical rationale

Daniel David Palmer, the founder of chiropractic, stated that the science, art and philosophy of chiropractic comprise the three essential areas of knowledge for the profession. Chiropractic philosophy has been a significant component of the profession’s culture [40]. Included in this philosophy is the subluxation, which was constructed as a legal tactic that allowed chiropractors to continue to treat patients without the required license to practice medicine [41]. The role of philosophy in chiropractic has been hotly debated [42]. The philosophical underpinnings of chiropractic have centered around the nervous system and, in more recent times, the effect of the vertebral subluxation complex on the nervous system’s functional role in other organ systems [43].

The philosophical rationale argues that the subluxation is derived by philosophical deduction from the major premise of universal intelligence and formulates the 33 principles [44]. This point argues that the subluxation is a true entity that impedes the expression of the metaphysical construct of innate intelligence and that the nervous system expresses such intelligence [45]. Thus, it is stated that the subluxation impedes upon this fuller expression and that correction of the subluxation brings forth the fullest expression of innate intelligence. While the biological plausibility of this paradigm is still a contentious topic, the claim that a vertebral subluxation has any causal effect on health has been refuted [24]. Furthermore, as Keating et al. explained, “when the speculative nature of a hypothesis or hypothetical construct is not made obvious, an otherwise acceptable proposition becomes a dogmatic claim. Such is the history of subluxation in chiropractic**”** [28].

One would expect subluxation to be mentioned in a beginning course, such as history of chiropractic. This study determined that such a term was commonly mentioned in principles and practice of chiropractic courses. We found of the US institutions that use the term subluxation (88%) the use ranged between 1 to 3 times in the course descriptions. Using the same methodology, it was found that of non-US programs, 26% (*n* = 5) used the term in course descriptions under principles and practice. This indicates that non-US programs place less emphasis on the term. Whether or not non-US programs merely mention the term as an appendage of past chiropractic history is unknown.

One must therefore question why this dogmatic philosophy is still maintained within chiropractic curricula. More than a decade ago, Wyatt el al [46] proposed that all chiropractic colleges adopt an evidence-based curriculum – a goal which to date has not been attained, as demonstrated by the continued use of subluxation in courses found in this study. This raises an important question: will patients in an exclusively subluxation based model receive optimal diagnoses and treatments? Croft et al. [47] stated that clinical practice should focus on “improving outcomes for patients in their total biological, psychological, and social environment and away from an exclusive and narrow focus on underlying disease as the determinant of outcome.” A practice that is exclusively subluxation based, thus driven by the doctor’s belief in the subluxation, cannot be evidence based. Such a practitioner will disregard important comorbidities that have been identified through empirical evidence that go beyond the subluxation and yet are amenable to care provided by a patient centered, evidence based practice chiropractors. Rather than clinging to an outdated construct, providers should implement a more patient-centered model, which has been shown to yield better outcomes [48]. This may help counter the public perception of chiropractors as the least ethical and honest healthcare profession [49]. Busse et al. [50] described how an unorthodox minority in chiropractic seems to hold powerful influence on orthopedic surgeons’ negative attitudes toward chiropractic. McGregor et al. [14] determined that this minority most strongly aligned with the statement: “chiropractic subluxation as an obstruction to human health” and were less likely to use evidence based treatment choices or guideline based radiography, and tended toward unorthodox vaccination attitudes. Likewise, Bussieres et al. found that graduates of colleges that most often used the term subluxation in their curricula, as reported by Mirtz and Perle [19], were more likely to take early and inappropriate spinal radiographs [51]. In addition, chiropractic educators and educational institutions have an obligation to provide non dogmatic, evidence based courses for future practitioners [46].

### Subluxation construct – Technical rationale

The technical rationale notes that the subluxation is a correctable and treatable biomechanical lesion via spinal manipulation for the treatment of many health afflictions [15, 24, 52]. The use of anecdotal evidence is used to demonstrate that such a construct exists. Many technique systems using either palpation and/or radiographic modalities have claimed to detect the subluxation. Many of these various systems of detection contend that their proscribed systems are superior forms of detecting and correcting the subluxation [53]; however these methods lack reliability [54].

This study identified the technical rationale by searching for the term subluxation in technique courses. Among US programs, 73.6% (*n* = 14) mentioned the term subluxation in technique courses. They used the term subluxation in course descriptions ranging from two times to as high as 11 times. Mirtz and Perle [19] discovered an average of 2.82 of technique courses included subluxation in their course descriptions, while this study found it increased to 3.83.

### Subluxation construct – Legal rationale

The fourth rationale used by adherents of subluxation rhetoric is the legal one, as seen in the many state practice acts [28, 55–57]. We realize that the concept of the subluxation may be enshrined in enabling registration for chiropractors within a jurisdiction. The only study we are aware of that investigates the scope of practice for chiropractic was by Chang [57], who noted that in Michigan various therapeutic procedures are permitted if they “relate to the subluxation complex.” A search of the (US) Federation of Chiropractic Licensing Boards’ Directory [58] revealed that of the 53 US jurisdictions (50 states, District of Columbia, Puerto Rico and US Virgin Islands), 13 mentioned subluxation. The only non-US jurisdiction included in the directory which mentioned subluxation in the licensing standards was from New Zealand, but few regulatory details are available outside of the US. We are aware that the General Chiropractic Council of the United Kingdom has stated that the subluxation complex is a historical and theoretical concept [59].

Our study did not seek to reveal how many courses use the term to satisfy legislative requirements controlling chiropractic practice. However, since 88% of US programs mention subluxation as opposed to 47.4% of non-US programs, certain legal requirements may be partly responsible for its use. However, politico-legal arguments do not confer scientific fact.

### Subluxation construct – Accreditation rationale

#### United States chiropractic programs

The US Council on Chiropractic Education (CCE) currently accredits 15 doctor of chiropractic programs (DCP) at 18 sites [60]. All US DCP are CCE accredited except for Keiser University. CCE also lists 16 members from all US DCP except Keiser University and Palmer College of Chiropractic, Florida. Subluxation was mentioned six times in the 2007 CCE Accreditation Standards [19]. The 2013 CCE Accreditation Standards [61] mentioned subluxation two times, first in the Preface*:*

*“…DCP education trains its graduates to…Assess and document a patient’s health status, needs, concerns and conditions with special consideration of axial and appendicular structures, including subluxation/neuro-biomechanical dysfunction.”*

and also in Meta-Competency 1: Assessment and Diagnosis:

*“…Performing case-appropriate physical examinations that include evaluations of body regions and organ systems, including the spine and any subluxation/neuro-biomechanical dysfunction, that assist the clinician in developing the clinical diagnosis.”*

The most recent accreditation standards, which go into effect January, 2018 [62] also use the term two times:

1. Meta-Competency 1, page 22: *“...Perform case-appropriate examinations that include evaluations of body regions and organ systems, including the spine and any subluxation/segmental dysfunction that assist the clinician in developing the diagnosis/es.”*

2. Outcomes, page 27: *“Students will be able to… Identify subluxations/segmental dysfunction of the spine and/or other articulations.”*

#### Non-US chiropractic degree programs

The Councils on Chiropractic Accreditation International list member institutions by affiliation [63]. Subluxation is not mentioned in accreditation standards of either the Council on Chiropractic Education Australasia (CCEA) [64] or the European Council on Chiropractic Education (ECCE) [65].

Canadian Federation of Chiropractic Regulatory and Educational Accrediting Boards (FCC) accredits Canadian Memorial Chiropractic College, Ontario, Canada and Université du Québec à Trois-Rivières, Quebec, Canada. Their website displayed 2011 Standards for Accreditation of Doctor of Chiropractic Programmes and mentioned subluxation twice, once on page 46 under Neuromusculoskeletal examination:

*“…the student must…understand and select methods for evaluating posture, biomechanical function, and the presence of spinal or other articular subluxation or dysfunction.”*

and, on page 55 under Chiropractic adjustment or manipulation skills:

*“…must select and effectively utilize palpatory and other appropriate methods to identify subluxations/joint dysfunctions of the spine and/or other articulation.”* [66]

The Federacion Latino Americo de Quiropractica (FLAQ) represents institutions in Mexico and South America: Feevale University, Brazil; Anhembi Morumbi University, Brazil; Universidad Estatal del Valle de Ecatepec, Mexico; Universidad Estatal del Valle de Toluca, Mexico; Universidad Central, Chile [67]. No specific accreditation standards were found on their website and email sent to FLAQ requesting accreditation details yielded no response.

Innes et al. [68] recently reviewed worldwide chiropractic accreditation standards and found the term subluxation appeared in the US CCE standards. They concluded the US CCE standard to identify subluxations is not evidence-based and should be questioned. One must therefore question why the US CCE continues to use the term. In another paper, the same authors [69] stated since inadequate physical examination skills lead to missed or delayed diagnoses, accrediting agencies should use unambiguous descriptive terms and prescribe each component of the physical examination competencies expected upon graduation. The authors concluded detecting subluxation was not based on adequate evidence or reproducible; therefore, its detection should not be included in a graduate’s competency. They recommended high quality evidence-based standards for educational institutions regarding patient care that help promote integration of chiropractic into mainstream healthcare. Other authors have concurred [5, 70].

#### Antagonists to subluxation rhetoric

Antagonists to the subluxation bring forth several rationales for critical questioning of the use of the subluxation terminology: the lack of scientific evidence, the inability for interdisciplinary function, and the cultural authority/professional credibility rationales.

The lack of scientific evidence for the subluxation appears to be the main reason for discounting such an entity. Keating et al. found that the subluxation construct lacks sufficient evidence to even reach the level of a theoretical construct and should be considered no more than pseudoscientific dogma [28]. Mirtz et al. found that the subluxation construct to have no epidemiological evidence thus not meeting the criteria for causation [24]. Other authors have questioned its applicability for clinical practice [71–73]. In fact, it can be sufficiently argued that clinical trials involving spinal manipulation are performed without even acknowledging a subluxation [74].

Our study found that several US programs used the term in courses other than principles and practice, technique and/or clinical philosophy courses. This study did find three prime examples from one institution that attempted to infuse subluxation rhetoric into such courses.

*Clinical Toxicology*

*This course is intended to enhance the student’s understanding of clinical pharmacology as related to disorders of the human organism, including vertebral subluxation.*

*Biochemistry I*

*This is a survey course intended to introduce the student to the chemistry and function of biomolecules with an emphasis on their role in human physiology. This course will lead to an understanding of the molecular basis underlying Physiology, Homeostasis, the effects of Subluxation and of its correction.*

*Genitourinary Diagnosis*

*The course is designed to give students a strong background in understanding the genitourinary system from a clinical chiropractic viewpoint. Genitourinary problems are varied and common in practice and are often related to Vertebral Subluxation Complex.*

Our literature search did not find any credible evidence that associates the chiropractic version of the vertebral subluxation complex to clinical toxicology, biochemistry and genitourinary diagnosis. The main problem with using subluxation in such a clinical context is known as the Ulysses syndrome. The Ulysses syndrome is defined a complication of false positive (healthy people diagnosed with disease) diagnostic test(s), diagnostic criteria, or clinical observations that are responsible for an aggressive diagnostic workup to elucidate what is, in reality, a non-disease state [75]. Such a non-diagnosis or non-clinical entity, i.e. subluxation as the exposure, is usually presented to the patient as the main clinical entity and has to be treated before the patient is allowed to return to his/her original state of health [73, 74]. In other words, the subluxation creates the Ulysses syndrome and is a side effect of unnecessary and inappropriate investigations or wrong interpretation of results. It thus creates the environment in which proponents of subluxation will utilize the term and worried patients will report such to medical professionals [76]. These types of patient encounters will create further marginalization of the profession based simply on use of the subluxation construct.

The chiropractic profession has not been very accepting of voices that call into question the chiropractic orthodoxy and its continued affinity for its zeitgeist. Ebrall [20] attempted to argue the unknown subluxation as a real entity by comparing it to x-ray. Such comparisons with actual science are intellectually dishonest in that x-ray can be measured as a wavelength and can be produced artificially and naturally. The subluxation does not have this capability. Skeptics of the subluxation essentially make the call to Hitchens’s variation of Occam’s razor: “What can be asserted without evidence can be dismissed without evidence” [77].

A valid criticism of the Mirtz and Perle [19] study was in the failure to assess course descriptions [20]. One of the main purposes of Mirtz and Perle’s paper was not simply in examining mentions of the term subluxation in college catalogs but how the term was used in course descriptions. Of all the course titles and descriptions examined, none were found to be critical of the subluxation in the Mirtz and Perle study. This study utilized the same methodology for all chiropractic programs examined and found that no course descriptions critically appraised the term subluxation.

There is little doubt about the perceived antagonism of mainstream medicine towards the chiropractic profession [78]. This study did not take into consideration other neuromusculoskeletal based professions such as physical therapy and athletic training and their use or non-use of subluxation. Yet one would be hard-pressed to imagine such professions using the term subluxation as an entity to treat. Even historically manipulation-based professions such as osteopathy fail to consider subluxation as a treatable entity via spinal manipulation. Thus, the subluxation lacks an interdisciplinary function, leaving the chiropractic profession marginalized. Such marginalization has caused the profession to not gain the needed cultural authority as a healing art [70].

### Limitations

An obvious limitation of the prior study by Mirtz and Perle was that it was restricted to North American chiropractic programs. While we attempted to get curricular data from all chiropractic programs worldwide, when this study was conducted, we were unable to obtain complete data for ten chiropractic programs. Further, there were three out of the ten signatories to the ICEC whose curricular information were unavailable. It would have been useful to determine if their curricula were congruent with the statements made by ICEC.

A major limitation of this study is that we are not able to determine what actually is taught. We can only report on the prevalence of the term subluxation in available course titles and descriptions. It is conceivable that a course without the term in the title or description may actually have significant content regarding subluxation. Likewise, a course with the term in the title or descriptions may be taught without ever talking about subluxation.

## Conclusions

The term subluxation was found in all but two US chiropractic course catalogues. The term was mentioned over eight times more frequently in US than non-US course catalogues. Similarly, subluxation was found greater than nine times more frequently in US course descriptions than in non-US descriptions.

US and Canadian accreditation standards still use the term. This contrasts with no mention in the standards of Australasian or European Council on Chiropractic Education member institutions.

Ten of the 28 international chiropractic institutions (35.7%) have joined ICEC, which published a position statement of Clinical and Professional Chiropractic Education. This statement included the following regarding the subluxation:

*“The teaching of vertebral subluxation complex as a vitalistic construct that claims that it is the cause of disease is unsupported by evidence. Its inclusion in a modern chiropractic curriculum in anything other than an historical context is therefore inappropriate and unnecessary.”* [38]. The ICEC members include Macquarie University (Australia); Murdoch University (Australia); Syddansk Universitet Odense (Denmark); Institut Franco-Europeen de Chiropratique (France); International Medical University (Malaysia); Durban University of Technology (South Africa); University of Johannesburg (South Africa); University of Zurich (Switzerland); AECC University College (United Kingdom); University of South Wales-Welsh Institute of Chiropractic (United Kingdom). To date no Western Hemisphere institutions have joined ICEC [38].

Unscientific terms and concepts should have no place in modern health care education, except perhaps in discussions with historical context. Unless these outdated concepts are rejected, the chiropractic profession and individual chiropractors will likely continue to face difficulties integrating with established health care systems and attaining cultural authority as experts in conservative neuromusculoskeletal health care.

## Abbreviations

BS: Bachelor of Science
CCEA: Council on Chiropractic Education Australasia
CCE: US Council on Chiropractic Education
DC: Doctor of Chiropractic
DCP: doctor of chiropractic program(s)
ECCE: European Council on Chiropractic Education
FCC: Canadian Federation of Chiropractic Regulatory and Educational Accrediting Boards
FLAQ: Federacion Latino Americo de Quiropractica
ICEC: International Chiropractic Education Collaboration
MS: Master of Science
PhD: Doctor of Philosophy
US: United States of America
